# Meta-analysis of Diurnal Transcriptomics in Mouse Liver Reveals Low Repeatability of Rhythm Analyses

**DOI:** 10.1177/07487304231179600

**Published:** 2023-06-29

**Authors:** Thomas G. Brooks, Aditi Manjrekar, Antonijo Mrcˇela, Gregory R. Grant

**Affiliations:** * Institute for Translational Medicine and Therapeutics, University of Pennsylvania, Philadelphia, Pennsylvania; † Department of Neuroscience, The University of Texas at Dallas, Richardson, Texas; ‡ Department of Genetics, University of Pennsylvania, Philadelphia, Pennsylvania

**Keywords:** transcriptomics, meta-analysis, circadian, phase distribution, mouse, liver

## Abstract

To assess the consistency of biological rhythms across studies, 57 public mouse liver tissue timeseries totaling 1096 RNA-seq samples were obtained and analyzed. Only the control groups of each study were included, to create comparable data. Technical factors in RNA-seq library preparation were the largest contributors to transcriptome-level differences, beyond biological or experiment-specific factors such as lighting conditions. Core clock genes were remarkably consistent in phase across all studies. Overlap of genes identified as rhythmic across studies was generally low, with no pair of studies having over 60% overlap. Distributions of phases of significant genes were remarkably inconsistent across studies, but the genes that consistently identified as rhythmic had acrophase clustering near ZT0 and ZT12. Despite the discrepancies between single-study analyses, cross-study analyses found substantial consistency. Running compareRhythms on each pair of studies identified a median of only 11% of the identified rhythmic genes as rhythmic in only 1 of the 2 studies. Data were integrated across studies in a joint and individual variance estimate (JIVE) analysis, which showed that the top 2 components of joint within-study variation are determined by time of day. A shape-invariant model with random effects was fit to the genes to identify the underlying shape of the rhythms, consistent across all studies, including identifying 72 genes with consistently multiple peaks.

Circadian or diurnal transcriptomic experiments study changes in expression of the entire transcriptome as a function of the time of day. Individual studies are limited by the difficulty and expense of gathering a sufficiently large number of samples to power the required statistical analysis. However, a growing number of such data are now available in public repositories. While an increasing number of transcriptomic meta-analyses are being performed (Brown and Peirson, 2018; Rau et al., 2014), meta-analyses examining the diurnal rhythm component of the transcriptome have so far been limited in scope (Keegan et al., 2007; Ness-Cohn et al., 2020; Brown et al., 2017) and have utilized primarily older microarray experiments (Li et al., 2018). Here we expand these investigations to a comprehensive review of RNA-seq experiments with particular attention to identifying and quantifying consistency of rhythmic behavior across studies.

While many available timeseries transcriptomics datasets investigate specific conditions and therefore contain non-comparable data, most include “control” conditions that are nominally identical. By allowing the inclusion of similar, but not identical, conditions (such as both nighttime-restricted feeding and ad libitum feeding in mice), a large set of “control” timeseries can be assembled. We investigate mouse liver—the most common mammalian tissue for circadian transcriptomics—by analyzing 57 RNA-seq timeseries studies containing 1096 samples identified from the Gene Expression Omnibus (GEO) repository. For each study, we started from the raw sequencing data which we processed in a uniform manner to obtain comparable quantifications from each timeseries. We assessed the consistency of these profiles using JTK_CYCLE analyses of the rhythmicity in each study.

We also performed additional meta-analyses across all studies. Meta-analyses often rely on random effects to capture differences across studies (Borenstein et al., 2010). We employ random-effect models, called shape-invariant models (SIMs; Wang et al., 2003), which perform non-linear curve fitting accounting for the differences between studies.

## Methods

### Data Collection

We searched the GEO repository using the GEOmetadb R package (Zhu et al., 2008) version 1.44.0 to identify mouse liver RNA-seq data containing references to the following terms: ZT, CT, zeitgeber, Bmal1, Cry1/2, Per1/2, Dbp, clock, constant conditions, entrain, darkness, circadian, or rhythm. The search was performed on GEO metadata collected 9 July 2021. The resulting accessions were assessed for the following: timeseries data of mouse liver samples; evenly spaced timepoints; at least 1 cycle of data; sampling interval of 6 h or faster; compatibility with our pipeline (excludes color-space data or datasets with large adapter sequences that did not align well); and at least 1 “control” condition. Control conditions were defined as meeting the following:

- Genotype: either wild-type mice (usually C57BL/6J) or a genotype used as control to another genotype (e.g., a *Cre*^+^ genotype)
- Feeding: either ad libitum food and water or night-restricted (ZT12-ZT14) feeding
- Sex: any allowed; if both male and female, sexes were separated into separate timeseries
- Light conditions: either light-dark (12 h: 12 h) (LD) or constant darkness (DD) conditions
- Interventions: none during days of sample collection; placebo or control treatments completed at least 24 h before sample collection

Some GEO records contain multiple matching experiments, such as 2 control timeseries for comparisons with 2 different genotypes. In such cases, the individual timeseries were treated as separate studies. Studies were labeled according to the first author and year of publication, or according to author and year made public on GEO if no publication was yet available.

### RNA-Seq Data Processing

A snakemake (v7.24.2) pipeline was developed to reproducibly process the data (Mölder et al., 2021). Reads were downloaded as sra files and converted to fastq format using the efetch, prefetch, and fastq-dump commands from edirect (v15.3) and sratoolkit (v2.11.0). Starting from sequencing reads, we quantified all samples using Salmon (Patro et al., 2017) (v1.4.0) to the GRCm38.75 Mus musculus transcriptome with the -k 31 index option. Salmon was run with the -lA —softclip —softclipOverhangs —seqBias —gcBias —reduceGCMemory —biasSpeedSamp 10 —posBias -p options as well as -g to quantify at the gene-level.

This generated read count estimates and transcripts per million (TPM) counts for each of 40,614 genes or transcripts.

### Quality Control

All data were manually inspected for consistent read depth and alignment statistics within each timeseries. Reported sex and tissue were confirmed by examining the expression of the sex-linked gene *Xist* and *Alb*, which is highly expressed in liver. Outlier data were identified by performing principal components analysis (PCA) on each timeseries individually and removing any samples that were at least 3 standard deviations from the mean in the first principal component. If any outliers were discarded, this process was repeated on the remaining samples until no more outliers were detected. In total, 14 samples were identified as outliers and removed from further analysis.

### Rhythmicity Testing

JTK_CYCLE (Hughes et al., 2010) was run on each timeseries TPM data with 24-h periods using MetaCycle 1.2.0 (Wu et al., 2016). Benjamini-Hochberg *q* values were computed from JTK_CYCLE *p* values after first dropping any genes that had mean read depth less than 2 reads across all samples in the timeseries. Dropped genes were assigned *q* = 1. After this filtering, the mean study had 16,214 genes.

Since the default for JTK_CYCLE is to use 20- to 28-h periods, we also ran it with that setting to determine if non-24-h genes were detectable. We selected 6 studies which had 8 or more timepoints per day (to improve ability to identify period) and were under the most common conditions (male, LD, ad libitum feeding). For each pair of studies, we computed Cramer’s *V* and Spearman *R* statistics comparing JTK periods on the genes significant in both (*q* < 0.05). These showed low consistency, with medians of *V* = 0.10, *R* = 0.07, and no pair achieving higher than *V* = 0.19 or *R* = 0.18. To check for consistently low-period genes, we searched for genes with period less than 24 in at least 4 of these 6 studies and with no period 24 or greater (when *q* < 0.05). Only 13 genes were identified. However, 100 random permutations of the period values gave a median of 16 identified genes by the same criteria. Due to this observed inconsistency in non-24-h period estimates, reported results were exclusively from using the fixed 24-h period, which also matches best-practice recommendations (Hughes et al., 2017).

To account for a greater variety of timeseries shapes, we further ran the BooteJTK method (Hutchison et al., 2018), which allows for asymmetric waveforms. The command used was BooteJTK-CalcP.py -f {expression.tpm.txt} -p ref_files/period24.txt -s /ref_files/phases_00-22_by2.txt -a ref_files/asymmetries_02-22_by2.txt -z 25 -r {num_reps} -R -x OUT, and if the study had either irregular numbers of replicates per timepoint or just a single timepoint, then the eJTK (Hutchison et al., 2015) software was first run and BooteJTK was run with the additional options -U -J {ejtk_output}.

### Comparison of Rhythms Across Studies

We ran the compareRhythms (Pelikan et al., 2022) method to test whether studies had differential rhythmicity. We used compareRhythms v1.0.1 in “voom” mode with a 24-h period on the quantified Salmon NumReads values. This reports 1 of 4 categories for each rhythmic gene as either loss, gain, same, or change in rhythmicity. In general, expression levels across 2 different studies are not comparable and so amplitudes are not comparable. We therefore were interested only in whether genes were rhythmic in both studies without regard for change in amplitude or phase, and so we grouped either “change” or “same” rhythmicity classifications as both meaning that the rhythm was present in both studies. We note that typically any comparison method such as compareRhythms would not be used to compare 2 timeseries acquired from entirely different experiments due to batch effects completely confounding the grouping. However, we are interested in measuring the size of the batch effects between these different studies and do not need to separate out batch effects from biological variation. In this way, the loss or gain of rhythmicity according to compareRhythms should be considered to include both batch effects and any actual biological variation.

### Robustness Score

For each gene, we computed its robustness score as the number of timeseries in which it was identified as significantly rhythmic according to JTK_CYCLE *p* < 0.05. With 57 total timeseries, the average non-rhythmic gene is expected to have a robustness score of about 3. With 40,614 genes measured and conservatively assuming that all were non-rhythmic, we can calculate that the Bonferroni-corrected *p* value of getting even a single gene with robustness score at least 13 is 0.024, from the survival function of the binomial distribution with *n* = 57, *p* = 0.05. Therefore, all genes with at least robustness score 13 are assumed to be genuinely rhythmic (in at least some studies) at a family-wise error rate of less than 0.05.

### PCAs

All PCAs were run on TPM data transformed by log (*x* + 0.01). PCA was run on the joined data from all timeseries to assess differences between studies. Furthermore, we ran PCA on each individual study to assess PCA performance as it would happen in any individual study. Finally, we ran a joint and individual variance estimate (JIVE; Lock et al., 2013) analysis, grouping within individual studies. We used 2 components of joint variation (as that typically is needed to capture the circular effect of time-of-day) and 1 component of individual variation in the JIVE analysis, to allow between-study variations.

### SIMs

SIMs were run to fit timeseries data to a periodic spline with a random-effects model allowing each timeseries in the meta-analysis to have different amplitudes, phases, and mean values (mesors). The random-effects structure compensates for the expectation that different studies have different values while still prioritizing consistency between studies. Fit splines then identify the consistent shape of curves throughout the day across all studies. Periods were fixed to 24 h, although the spline may also fit ultradian rhythms at harmonic periods (such as 12- or 8-h periods). The R package assist (v3.1.7) was used with the snm function (Wang et al., 2003). The convergence condition was set to the “PRSS” method with convergence criterion prec.out = 0.05. Values for lambda parameter were restricted to 10^−4^ to 10^3^ to prevent overfitting that happened at the default settings (10^−10^ to 10^3^). The SIM model was fit to each gene that had non-zero values in at least one-third of all samples across all studies, after outlier removal described earlier.

The *R*^2^ goodness-of-fit value was computed for each timeseries and each gene as (SS_total_ – SS_resd_) / SS_total_ where SS_total_ and SS_resid_ are the squared sum of differences from the mean value and from the model fit of the data, respectively. Due to the existence of random effects in the model, this is not guaranteed to be positive, unlike the *R*^2^ of a linear model. Nonetheless, larger values are indicative of better fits.

The fit for a gene was classified as rhythmic if it satisfied:

- Fit converged in at most 30 iterations,
- SD of the logAmp random-effect parameter is at most 3 (i.e., most timeseries should have amplitudes within a factor of 20 of the mean amplitude),
- funcDf < 15 (i.e., fitting at most 15° of freedom to the spline, considering that 23° of freedom is enough to fit each hour with an independent value),
- median_t > 2 (i.e., the median across time of the *t* statistic (difference from zero divided by standard error) is at least 2, so that the spline is significantly non-zero at most times), and
- median across all studies of *R*^2^ > 0.25.

To estimate the false discovery rate of these criteria, a 1-shot permutation test was performed, where the data within each study had timepoint labels permuted randomly. Permutations were done independently in each gene, but due to computational costs, only a single permutation was taken for each gene. In the permuted data, there were only 15 genes satisfying the rhythmicity criteria while in the original data, there were 2712 such genes. Therefore, we estimate a false discovery rate of less than 0.01.

Fits were classified as either monomodal symmetric, monomodal asymmetric, or multimodal. Symmetry was assessed by comparing the fit curve with all its cyclic mirror images (one reflected about every quarter hour). If all mirror images contained some points of at least 2 standard errors (determined by the pstd variable) different from the original curve, then the gene was classified as asymmetric. Therefore, fits symmetric about even 1 point would not be identified. To identify multimodal genes, we looked for at least 2 distinct peaks, meaning 2 points at least 2 standard errors from zero and with at least 1 point below zero between them in both clockwise and counterclockwise directions.

## Results

We identified 275 GEO records for consideration, of which 33 have evenly spaced time points in mouse liver with a resolution of at least 6 h and span at least 1 full day. Twelve additional GEO records that post-dated the search were identified during revisions and included. After selecting for approximate “control” conditions and separating out multiple studies within GEO records by experiment, condition, and sex, we obtained 57 studies containing 1096 samples, see Table 1. Starting from sequencing reads, we quantified all samples using Salmon (Patro et al., 2017), giving all data a consistent reference genome and annotation.

**Table 1.** Studies analyzed.

Name	GSE	PubMed	Sex	Light	Age (weeks)	Sample Count	Timepoints Per Cycle	Replicates	Cycles	Sequencing Type	Notes
Abe22	GSE199061	35999195	M	DD	8-24	12	6	2	1	SS PE PolyA	(Abe et al., 2022)
Acosta-Rodríguez22A	GSE190939	35511946	M	DD	26	24	6	2	2	SS SE PolyA	(Acosta-Rodriguez et al., 2022)
Acosta-Rodríguez22B	GSE190939	35511946	M	DD	82	24	6	2	2	SS SE PolyA	(Acosta-Rodriguez et al., 2022)
Astafev23	GSE216416		F	LD	21	17	6	2-3	1	US PE PolyA	
Aviram21	GSE171975	34968386	M	DD	12	48	6	4	2	SS SE 3prime	(Aviram et al., 2021)
Benegiamo18	GSE98042	29358041	M	LD	14	12	12	1	1	SS SE RiboZero	(Benegiamo et al., 2018)
Brooks22^a^	GSE115264	34724846	M	DD	16-24	25	6	4-5	1	SS PE PolyA	(Brooks et al., 2022)
Cajan16	GSE61775		M	LD	10-12	24	12	2	1	US PE PolyA	
Chaix19A^b^	GSE102072	30174302	M	LD	24	12	6	2	1	SS SE PolyA	(Chaix et al., 2019)
Chaix19B^c^	GSE102072	30174302	M	LD	24	11	6	1-2	1	SS SE PolyA	(Chaix et al., 2019)
Du14^d^	GSE57313	24867642	M	LD	12-24	12	6	2	1	US SE RiboZero	(Du et al., 2014)
Fader19^e^	GSE119780	31015483	M	LD	9-10	24	8	3	1	SS SE PolyA	(Fader et al., 2019)
Frazier22A^f^	GSE184303		M	LD	13-17	18	6	3	1	SS SE PolyA	(Frazier et al., 2022)
Frazier22B	GSE184303		M	LD	13-17	18	6	3	1	SS SE PolyA	(Frazier et al., 2022)
Gaucher19	GSE132103	31757851	M	LD	9-24	18	6	3	1	SS SE PolyA	(Gaucher et al., 2019)
Greenwell19A	GSE118967	30995463	M	LD	12-13	18	6	3	1	SS SE 3prime	(Greenwell et al., 2019)
Greenwell19B^g^	GSE118967	30995463	M	LD	12-13	18	6	3	1	SS SE 3prime	(Greenwell et al., 2019)
Guan20^h^	GSE143524	32732282	M	LD	8-12	24	8	3	1	SS PE PolyA	(Guan et al., 2020)
Hirako18	GSE109908	29805094	F	LD	10	12	4	3	1	SS SE PolyA	(Hirako et al., 2018)
Katsioudi22	GSE208768	36638184	M	LD	52-104	18	6	3	1	SS PE RiboZero	(Katsioudi et al., 2023)
Kinouchi18	GSE107787	30566858	M	LD	8	18	6	3	1	SS SE PolyA	(Kinouchi et al., 2018)
Koritala22	GSE214530		M	DD	9	24	8	3	1	US PE PolyA	
Koronowski22	GSE158600	34550736	M	LD	8-12	36	6	6	1	SS PE RiboZero	(Greco et al., 2021)
Lahens15	GSE40190		M	DD	6	8	4	1	2	US PE PolyA	(Lahens, 2014)
Levine20	GSE133989	32369735	M	LD	32	19	6	3-4	1	SS SE PolyA	(Levine et al., 2020)
Li19A	GSE113745		M	LD	16	12	6	2	1	SS SE PolyA	
Li19B	GSE113745		M	LD	76	12	6	2	1	SS SE PolyA	
Li20^i^	GSE133342	32160860	M	DD	6	6	6	1	1	US PE RiboZero	(Li et al., 2020)
Manella21^j^	GSE159135	34059820	M	LD	12-16	24	12	2	1	SS SE 3prime	(Manella et al., 2021)
Mekbib22A	GSE182834	35789210	M	LD	20	8	8	1	1	SS PE PolyA	(Mekbib et al., 2022)
Mekbib22B	GSE182834	35789210	F	LD	20	8	8	1	1	SS PE PolyA	(Mekbib et al., 2022)
Mermet18	GSE101423	29572261	M	LD	8-12	6	6	1	1	SS SE PolyA	(Mermet et al., 2018)
Mezhnina22	GSE211975	36161962	F	LD	21	18	6	3	1	US PE PolyA	(Mezhnina et al., 2022)
Mortimer21^k^	GSE117134	34036284	F	LD	8-12	18	6	3	1	SS PE RiboZero	(Mortimer et al., 2021)
Morton20	GSE151565	36265442	M	LD	26	77	8	5-6	1.625	SS PE PolyA	(Wang et al., 2022)
Pan20^l^	GSE130890	31935211	M	DD	8-12	48	12	2	2	SS PE PolyA	(Pan et al., 2020)
Petrus22	GSE196430	35767612	M	LD	8-12	18	6	3	1	SS PE PolyA	(Petrus et al., 2022)
Quagliarini19A	GSE108688	31706703	M	LD	17-18	17	6	2-3	1	US PE PolyA	(Quagliarini et al., 2019)
Quagliarini19B^b^	GSE108688	31706703	M	LD	17-18	18	6	3	1	US PE PolyA	(Quagliarini et al., 2019)
Quagliarini19C	GSE108688	31706703	M	LD	17-18	17	6	2-3	1	SS PE PolyA	(Quagliarini et al., 2019)
Quagliarini19D^b^	GSE108688	31706703	M	LD	17-18	17	6	2-3	1	SS PE PolyA	(Quagliarini et al., 2019)
Rubio-Ponce21	GSE125867	33937766	M	LD	8-9	18	6	3	1	US SE PolyA	(Rubio-Ponce et al., 2021)
Sinturel17A	GSE73552	28475894	M	LD	12-14	48	12	4	1	SS PE RiboZero	(Sinturel et al., 2017)
Sinturel17B^g^	GSE73552	28475894	M	LD	12-14	24	6	4	1	SS PE RiboZero	(Sinturel et al., 2017)
Stubblefield18	GSE105413	29386110	M	LD	9-12	32	8	4	1	SS SE RiboZero	(Stubblefield et al., 2018)
Trott18	GSE36871	29300726	M	LD	12-24	12	6	2	1	US SE PolyA	(Trott and Menet, 2018)
Weger19A	GSE114400	30344015	M	LD	15-16	12	6	2	1	SS PE RiboZero	(Weger et al., 2019)
Weger19B	GSE114400	30344015	F	LD	15-16	12	6	2	1	SS PE RiboZero	(Weger et al., 2019)
Weger21A	GSE135898	33452134	M	LD	9-14	12	6	2	1	SS PE PolyA	(Weger et al., 2021)
Weger21B	GSE135875	33452134	M	LD	9-14	12	6	2	1	SS PE PolyA	(Weger et al., 2021)
Weger21C	GSE135898	33452134	M	LD	9-14	12	6	2	1	SS PE PolyA	(Weger et al., 2021)
Wu19	GSE138019	31875550	M	LD	16-24	6	6	1	1	SS SE RiboZero	(Wu et al., 2019)
Wu23	GSE195456		M	LD	8-52	18	6	3	1	US PE PolyA	(Wu et al., 2023)
Xin21^i^	GSE150380	33889826	F	LD	9	28	6	4	1.17	SS PE RiboZero	(Xin et al., 2021)
Yang16A^m^	GSE70497	26843191	M	DD	16-24	24	6	4	1	US PE PolyA	(Yang et al., 2016)
Yang16B	GSE70499	26843191	M	LD	6-14	9	6	1-2	1	SS PE PolyA	(Yang et al., 2016)
Yang16C	GSE70499	26843191	F	LD	6-14	9	6	1-2	1	SS PE PolyA	(Yang et al., 2016)
Zhang14	GSE54651	25349387	M	DD	6-7	8	4	1	2	SS PE PolyA	(Zhang et al., 2014)

Abbreviations: GSE = Gene Expression Omnibus series identifier; M = male; F = female; DD = constant darkness conditions; SS = strand-specific; PE = paired-end; SE = single-end; LD = 12-12 h light: dark conditions; US = unstranded; 3prime = 3-prime specific sequencing; PolyA = poly(A) selected sequencing; RiboZero = RiboZero rRNA depleted sequencing; SPF = specific pathogen free; GF = germ free; NRF = night-restricted feeding.

Studies were included if they had in vivo mouse liver RNA-seq, had at least 4 evenly spaced timepoints per day, included data from “control” conditions (meaning no major interventions or non-wild-type genotypes, see “Methods”), and the data were available on GEO and were compatible with our pipeline. Replicates column denotes the mean number of biological replicates at each collection time. Cycles denotes the number of 24-h periods measured, counted such that, for example, samples every 4 h for 6 timepoints would be 1 cycle (even though the ZT24 time would not be included until the seventh timepoint). Sequencing Type column details the type of RNA-seq performed. Additional study information, when necessary, in footnotes.

a *Bmal1*^fl/fl^.

b High fat diet.

c Night-restricted feeding; high fat diet.

d *AlbCre-ERT2*; tamoxifen treated.

e Sesame oil gavage.

f Bmal1^fl/fl^; specific pathogen free.

g Night-restricted feeding.

h Rev-erbα^fl/fl^; Rev-erbβ^fl/fl^.

i Six weeks of constant darkness.

j *Alb-Cre*^+^.

k *Alfp-Cre*^−^/tg.

l *XBP1*Flox.

m *Bmal1*^fl/fl^; tamoxifen treated.

To better assess differences between studies, we note that the most common study design is male mice, LD lighting, and ad libitum feeding of standard chow without any interventions. There are 20 such studies which are therefore highly comparable, differing primarily in age or factors that are often unreported (such as housing).

### Technical Factors Dominate Biology and Study Design

To assess the overall similarities of studies, we performed a PCA on the log-scaled transcripts per million (log TPM) expression values. These revealed that the largest differences between studies were driven by technical factors in the sequencing (Suppl. Fig. S1). In particular, the 3 timeseries which sequence only the 3’ end of the transcript were outliers. Similarly, RiboZero versus PolyA-selected libraries are clearly distinct. Smaller differences were observed between stranded versus unstranded sequencing and paired-end versus single-end sequencing. Differences from biological factors, such as male and female, were smaller than theses technical differences.

### Phases of Core Clock Genes Are Consistent

**Figure 1.**
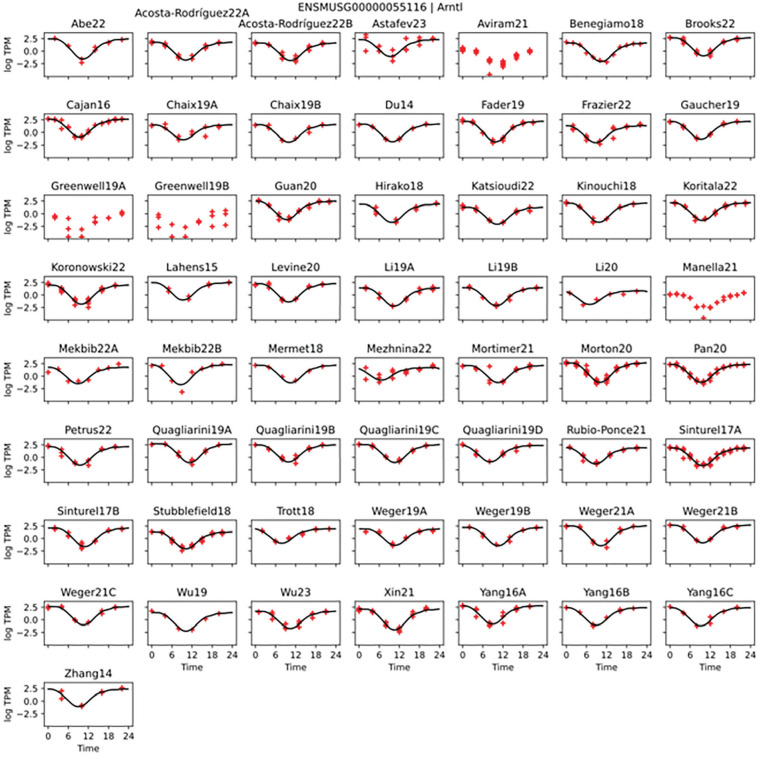
Core clock genes are highly consistent. Data from 57 mouse liver RNA-seq circadian timeseries were processed. *Arntl* (*Bmal1*) gene quantified log transcripts per million (TPM) in red. Time 0 corresponds to lights on (ZT0) or to the equivalent CT0, depending on study. Shape-invariant models curve fit in black (with 4 studies excluded from the fits due to using an uncommon sequencing methodology). See other core clock genes in Supplementary Figure S2.

Using the reported ZT/CT times for each study, we plotted time-course profiles of the TPM expression values of 7 core clock genes (*Arntl, Cry1, Cry2, Per1, Per2, Nr1d1, and Clock*) (Ko and Takahashi, 2006) for each study and found that the peaks and troughs for these genes are well aligned across all timeseries (Figure 1, Suppl. Fig. S2). While the amplitudes vary moderately, the plots reveal remarkable consistency in phase and period across all studies despite the differences in study designs.

### Single-study Analyses Have Low Consistency

**Figure 2.**
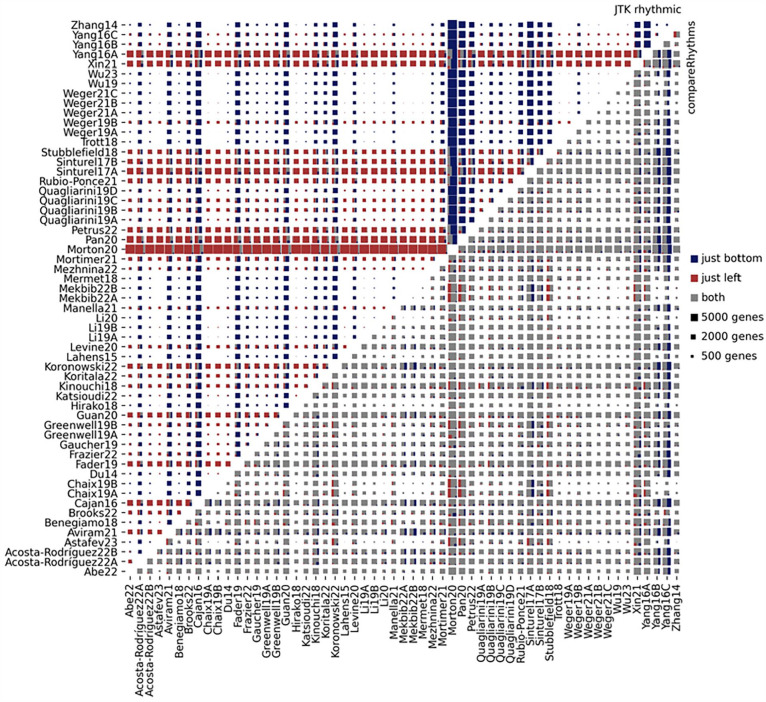
Rhythmic gene overlaps in JTK and compareRhythms. Size of genes rhythmic common to both studies (in gray), unique in the row label study (red), or unique in the column label study (blue) shown for both JTK_CYCLE (above diagonal) and compareRhythms (below diagonal). JTK_CYLCE was run on each timeseries, and results were compared and genes significant at Benjamini-Hochberg *q* < 0.05 were considered rhythmic. Next, compareRhythm was run on every pair of studies which classified each gene as either loss, gain, change, or same rhythm between the 2 studies. Since expression values differ between studies making amplitude changes inestimable, we considered genes to be rhythmic in both studies (possibly of differing amplitudes or phases) if they were identified as either same or change.

We ran JTK_CYCLE (Hughes et al., 2010) (JTK) on each timeseries separately. We then compared the results of the datasets in several ways. First, we selected the significantly rhythmic genes at *q* < 0.05 identified by JTK and compared the overlaps of these lists between different studies (Figure 2) and likewise for BooteJTK significant genes (Suppl. Fig. S3). Differences of significant gene lists between studies was large for both JTK and BooteJTK. Gene lists were compared by their Jaccard index (i.e., the size of the intersection divided by the size of the union of significant genes in both datasets). The median Jaccard index was just 2% for JTK and 12% for BooteJTK; the highest indexes were 47% and 60% for JTK and BooteJTK, respectively.

Notably, sample size seemed to be more important for large overlap between studies than was study design. For example, 2 of the largest studies, Morton20 and Pan19, differ in lighting conditions (12-h LD and DD, respectively) but had the second highest JTK overlap of 47%. This emphasizes the importance of having enough samples to obtain repeatable results. Studies from the same laboratories showed only modestly higher agreement with each other than with other similar studies. Sample count was overall less important in the BooteJTK analysis where the highest overlap went to 2 studies (Quagliarini19A and Mezhnina22) that had only 18 and 17 samples and differed in the sex of the mice studied.

In contrast, the compareRhythms analysis directly compares the datasets and found relatively low levels of identifiable genes with rhythm in one study but not the other study (Figure 2). The Jaccard index from compareRhythms had a median of 89%, and many studies achieved 100% matches (no genes identified as loss or gain of rhythm between the 2 studies). This indicates that although the identified list of *q* < 0.05 genes from any study has low replicability, the evidence of genuinely distinct rhythmicity across studies is more limited.

**Figure 3.**
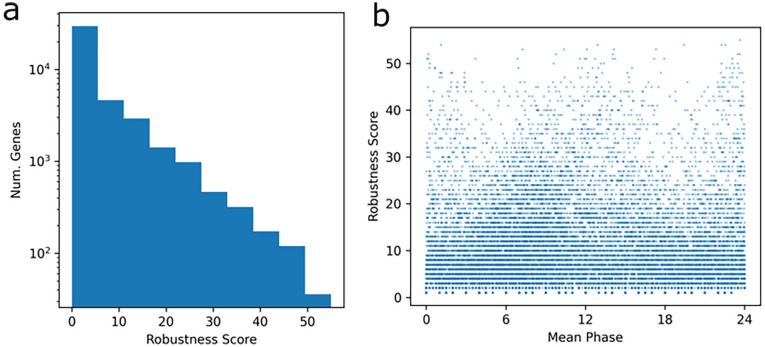
Robustness of genes. JTK_CYCLE results were summarized across all studies to identify genes that were highly consistent. The robustness score was computed as the number of studies in which the genes had JTK_CYCLE *p* value under 0.05. Correcting for the number of studies and genes, robustness scores of 13 or higher have *p* < 0.05 if the gene is not rhythmic in any study. (a) Number of genes by robustness score. (b) Plot of robustness score by mean (across studies in which JTK_CYCLE *p* < 0.05) phase for each gene. Genes with the highest robustness scores cluster in phase near ZT0/ZT24 or ZT12.

To identify the genes consistently identified as rhythmic across studies, we employed a simple voting-counting metric. We defined the *robustness score* of a gene as the number of studies in which it was identified to be significantly rhythmic according to JTK (Figure 3a) at *p* < 0.05, out of a maximum possible score of 57. We identified 5222 genes with a robustness score of at least 13, which has a *p* < 0.05 chance of giving even 1 false positive gene, see “Methods.” Moreover, 525 genes reached a robustness score of 35 or more and therefore exhibit rhythmic behavior across many studies, see Supplementary Table S1. High robustness genes cluster around ZT0 and ZT12 in acrophase (Figure 3b).

### Phase Distribution Consistency

**Figure 4.**
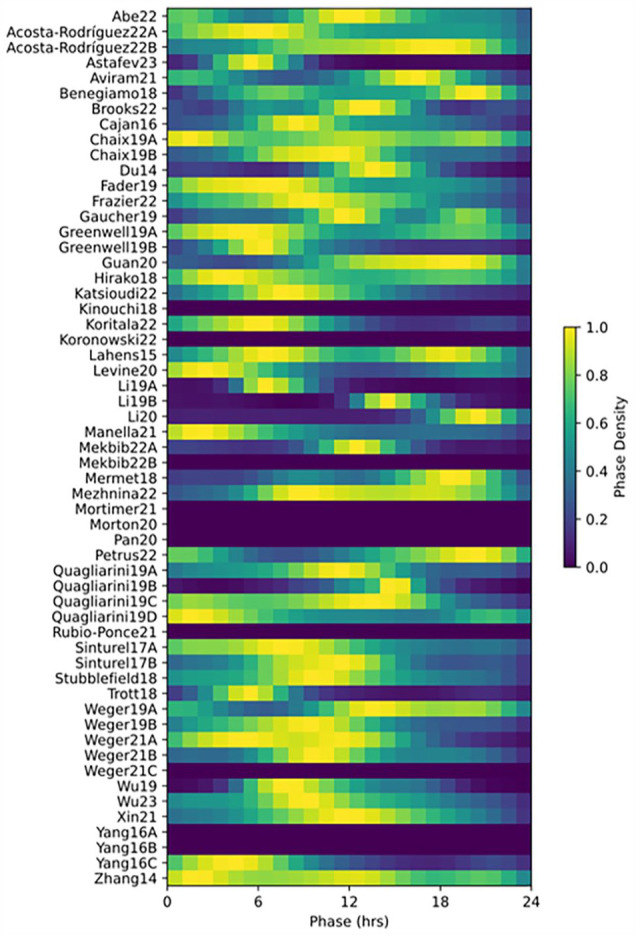
Consistency of phase distributions. JTK_CYLCE was run on each timeseries, and results were compared. Distributions of phases among genes identified rhythmic by JTK (at a Benjamini-Hochberg *Q* value < 0.05), showing notable inconsistency between studies. Distributions are normalized to peak 1, with the total number of genes identified as rhythmic shown in separate column. Phase distributions skipped in studies with fewer than 10 rhythmic genes.

We next compared consistency of the distribution of phases of identified rhythmic genes. These distributions are routinely used to summarize overall activity in the transcriptome. However, these showed remarkable inconsistency across studies (Figure 4, Suppl. Fig. S4). While many studies showed preferential clustering of phases near particular times of day, the locations of these clusters differed substantially between studies. These differences are not explained by factors such as 12-h LD versus DD conditions; for example, Morton20 and Sinturel17A have the same conditions, large sample sizes, and moderately high overlap in identified rhythmic genes, but have almost opposite peaks in their phase distributions. However, when a single set of rhythmic genes is chosen for all studies, then the phase distributions are considerably more consistent, with all showing a peak near ZT12 (Suppl. Fig. S5). In contrast to phase, amplitude distributions were more consistent across studies (Suppl. Fig. S6).

### Joint PCA (JIVE) Identifies Consistent Rhythmicity Across Studies

**Figure 5.**
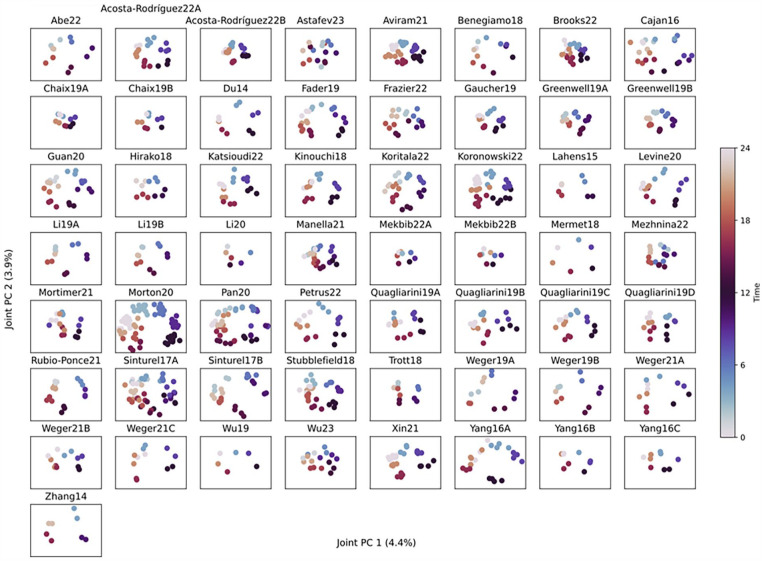
Joint and individual variation estimation (JIVE) reveals consistent rhythmicity across studies. A JIVE analysis was used to determined loadings of genes that have consistently high variance within each study (regardless of between-study differences). The identified 2 joint variance components are plotted in each timeseries, showing consistent separation by time-of-day. All plots use the same gene loadings.

To examine consistent factors of variance across the studies, a JIVE analysis was performed (Lock et al., 2013). While PCA on aggregated data from multiple studies identifies variance primarily between studies (Suppl. Fig. S1a), JIVE divides the within-studies variance into a joint component (common to all studies) and individual components (distinct for each study). JIVE does this by finding *n* *+* *m* principal components to capture as much variance of each study as possible, where the first *n* components are the joint components and are the same for all studies, while the last *m* are individual components and are allowed to be different for each study. Specifying *n* = 2 and *m* = 1, we found that the joint variance components capture the rhythmicity in factors that cleanly and consistently separate timepoints across all studies, see Figure 5. These 2 joint variance components together account for 8.3% of the within-study variance. In contrast, PCA run on individual studies gives inconsistent results, see Supplementary Figure S7.

### SIMs Reveal the Shape of the Transcriptome Rhythms

SIMs fit a flexible curve to multiple independently measured studies, assuming there is a consistent shape across studies (Wang et al., 2003). Moreover, they allow for random-effects modeling by allowing each measured study to have differences in phase, amplitude, and mid-level (mesor). Applying this to our dataset allows the pooling of information from all studies to determine more accurately the underlying consistent shape of the rhythms within each study. We identified 2712 significantly rhythmic shapes (see “Methods”). Core clock genes were well-identified (Figure 1a, Suppl. Fig. S2).

To visualize the overall spectrum of diurnal profiles, we normalized all SIM fits to have amplitude 1 (max to min) and to peak at the same time. This allows us to consider profile shapes without considering amplitude or phase. These normalized shapes were sampled every 15 min, yielding, for each rhythmic gene, a vector of 96 values each ranging from 0 to 1. To group similar shapes together, a t-SNE dimension-reduction was performed on these normalized shapes, and the results were plotted (Suppl. Fig. S8a). Genes with significant fits were classified to either as monomodal with symmetric (*n* = 1349) or asymmetric (*n* = 1291) peaks, or as multimodal (*n* = 72). This emphasizes the diversity of profiles present in the transcriptome, although the most common shapes are similar to the classic cosine curve.

### Genes With High Variability in Phase

Next, we considered the set of rhythmic genes which displayed the largest variability in their phases between studies. We hypothesized that these genes will be sensitive to external factors. Using the identified rhythmic SIM fits, the mean variability in phase was 1.11 h, see Supplementary Figure S8b, determined by transforming the SIM phi value to hours by an inverse logistic function to approximate the standard deviation of phases. We identified 187 genes with at least 2 h phase variability. Pathway analysis identified no pathways enriched for high phase variability.

### Consistent Non-rhythmic Genes

We searched for genes that were consistent across studies and time by requiring that for each gene, the mean TPM was at least 1, the standard deviation was at most half the mean TPM, and that JTK_CYCLE *q* value was at least 0.05, as well as not having a rhythmic SIM fit. We chose these criteria to require high expression, low variance, and no detectable time-of-day dependence. A total of 98 genes met these criteria, see Supplementary Table S2. These genes represent candidate lists of “housekeeping” genes that repeatedly have minimal time varying across a wide selection of studies and have high expression values. The commonly used housekeeping gene Gapdh was not on the list, due to having standard deviation of TPM over half of its mean TPM in 3 studies, as well as being significantly rhythmic by JTK in 4 studies (and close to significant in several others). This demonstrates that these criteria are stringent and identify only highly consistent genes in mouse liver samples and may be useful as reference non-cyclic genes.

## Discussion

Meta-analyses of transcriptomics are becoming increasingly popular as more datasets become publicly available, and the importance and practicality of such studies are increasing. Here, we perform the first large-scale meta-analysis of circadian or diurnal timeseries transcriptomics that we are aware of. We found that analyses restricted to single studies consistently capture rhythms in core clock genes. However, substantial variability outside of the core clock between studies highlights the limitations of single-study datasets. Variability was such that differences from light condition (LD vs. DD) were smaller than between-study variation, with low sample counts contributing to this. Despite this, directly comparing rhythms across different studies reveals a much smaller discrepancy, suggesting that the large differences between single-study analyses are driven by statistical variation more than genuinely distinct rhythmic patterns.

Previous studies have found low overlaps in the sets of genes rhythmic at the protein level and those rhythmic at the transcript level. Our low observed overlap between transcript-level studies indicates that these may give underestimates of the true overlap, particularly when comparing across different sets of mice, as some studies have done (Robles et al., 2014).

Phase distributions showed marked differences between studies, even those with the largest sample counts or highest temporal resolutions and under the same conditions. This suggests caution while interpreting phase distribution plots from individual studies. Since phase distributions were considerably more consistent when a fixed set of genes was compared across all studies, the differences in phase distributions may be driven by differences in the set of genes identified as rhythmic rather than the phases of individual genes.

In contrast, meta-analysis identifies rhythmic factors that are consistent across many studies. A JIVE analysis demonstrates that times accounts for 8.3% of the within-study variance by identifying 2 components of variation common to all studies. These components give highly consistent results in all studies, demonstrating that despite their differences in single-study analyses, all studies contain a large underlying component of consistency.

Individual studies have limitations in resolution and replication necessary to confidently identify the shape of time-course expression profiles at the transcriptome-wide scale. By pooling data from all studies in a SIM analysis, we obtained reliable curve fits that do not overfit to the noise in any individual study. These allow us to observe that approximately half of all rhythmic genes in mouse liver have asymmetric patterns, and a small number show multimodal patterns, even under LD conditions. Since many analysis methods make the assumption of symmetry, such as JTK_CYCLE (Hughes et al., 2010) and cosinor (Cornelissen, 2014), this informs the choice of alternative methods that have fewer assumptions of shape, such as RAIN (Thaben and Westermark, 2014) and BooteJTK (Hutchison et al., 2018).

We observed large differences from sequencing parameters (such as rRNA depletion method or strand specificness), which were not always well-described in the corresponding publications. We therefore recommend more prominently describing such parameters in future studies.

One limitation of this study is the inclusion of data under multiple biological conditions. This likely decreases the amount of observed consistency between studies. However, even restricting to a subset of studies with highly consistent designs (male mice, LD lighting, and ad libitum feeding of standard chow), we find substantial inconsistencies on single-study analyses. Moreover, by including multiple biological conditions, results from SIM and JIVE analyses will capture effects that are consistent across those conditions and therefore of broad interest. Since these meta-analytic methods naturally account for differences between the individual studies, the inclusion of multiple study designs and conditions should not compromise the results. A further limitation is the small number of studies including female mice, which are known to be underused in circadian model animal studies (Obodo et al., 2023).

## Conclusion

When a gene is found to be rhythmic in one data set and not in another, it can be due to technical factors that influence the statistical power to detect, or it can reflect the true biology—the gene in fact was rhythmic in one set of animals and not in the other. Datasets which compare one condition with a control condition wish to identify effects that are driven by the condition. However, the results of this meta-analysis indicate that a significant amount of variation can be due to variation in the baseline themselves. This then raises the question of the proper interpretation of differences identified between condition and control experiments.

We have observed from this study that rhythm detection in transcriptomics is limited by sample counts, by demonstrating considerable variability in “control” conditions across studies in the identified rhythmic genes as well as in distributions of phase among those genes. Meta-analysis is a key tool for researchers to arrive at consensus that remains under used in circadian transcriptomics despite a growing wealth of data available. Meta-analysis also answers key questions that are not answerable in any individual study no matter the sample count, such as how similar would our observations be if someone else repeated the experiment? Effects that exist in a single study may be of limited interest, even those that pass statistical significance. Use of “mega-analysis” (Lin and Zeng, 2010), where the original data from all studies is analyzed instead of just summary statistics, further allows exploring new questions, such as robustly identifying the shape of the temporal profile of these genes.

## Supplemental Material

sj-docx-1-jbr-10.1177_07487304231179600 – Supplemental material for Meta-analysis of Diurnal Transcriptomics in Mouse Liver Reveals Low Repeatability of Rhythm Analyses Click here for additional data file. Supplemental material, sj-docx-1-jbr-10.1177_07487304231179600 for Meta-analysis of Diurnal Transcriptomics in Mouse Liver Reveals Low Repeatability of Rhythm Analyses by Thomas G. Brooks, Aditi Manjrekar, Antonijo Mrccˇela and Gregory R. Grant in Journal of Biological Rhythms

sj-txt-2-jbr-10.1177_07487304231179600 – Supplemental material for Meta-analysis of Diurnal Transcriptomics in Mouse Liver Reveals Low Repeatability of Rhythm Analyses Click here for additional data file. Supplemental material, sj-txt-2-jbr-10.1177_07487304231179600 for Meta-analysis of Diurnal Transcriptomics in Mouse Liver Reveals Low Repeatability of Rhythm Analyses by Thomas G. Brooks, Aditi Manjrekar, Antonijo Mrccˇela and Gregory R. Grant in Journal of Biological Rhythms

sj-txt-3-jbr-10.1177_07487304231179600 – Supplemental material for Meta-analysis of Diurnal Transcriptomics in Mouse Liver Reveals Low Repeatability of Rhythm Analyses Click here for additional data file. Supplemental material, sj-txt-3-jbr-10.1177_07487304231179600 for Meta-analysis of Diurnal Transcriptomics in Mouse Liver Reveals Low Repeatability of Rhythm Analyses by Thomas G. Brooks, Aditi Manjrekar, Antonijo Mrccˇela and Gregory R. Grant in Journal of Biological Rhythms
