# Supplementary material for: Meta-analysis of Diurnal Transcriptomics in Mouse Liver Reveals Low Repeatability of Rhythm Analyses
Source: J Biol Rhythms. 2023 Jun 29;38(6):556–70. doi: 10.1177/07487304231179600 (PMC10615793; doi:10.1177/07487304231179600)
Supplement: sj-docx-1-jbr-10.1177_07487304231179600 – Supplemental material for Meta-analysis of Diurnal Transcriptomics in Mouse Liver Reveals Low Repeatability of Rhythm Analyses [file sj-docx-1-jbr-10.1177_07487304231179600.docx]

Meta-analysis of diurnal transcriptomics in mouse liver reveals low repeatability of rhythm analyses

Thomas G. Brooks^1^, Aditi Manjrekar^2^, Antonijo Mrčela^1^, Gregory R. Grant^1,3^

1. Institute for Translational Medicine and Therapeutics, University of Pennsylvania, Philadelphia, PA 19104, USA
2. Department of Neuroscience, University of Texas at Dallas, Richardson, TX 75080, USA
3. Department of Genetics, University of Pennsylvania, Philadelphia, PA 19104, USA

Corresponding author: Thomas G. Brooks thobr@sas.upenn.edu

# Supplemental

## Supplemental Figures

| 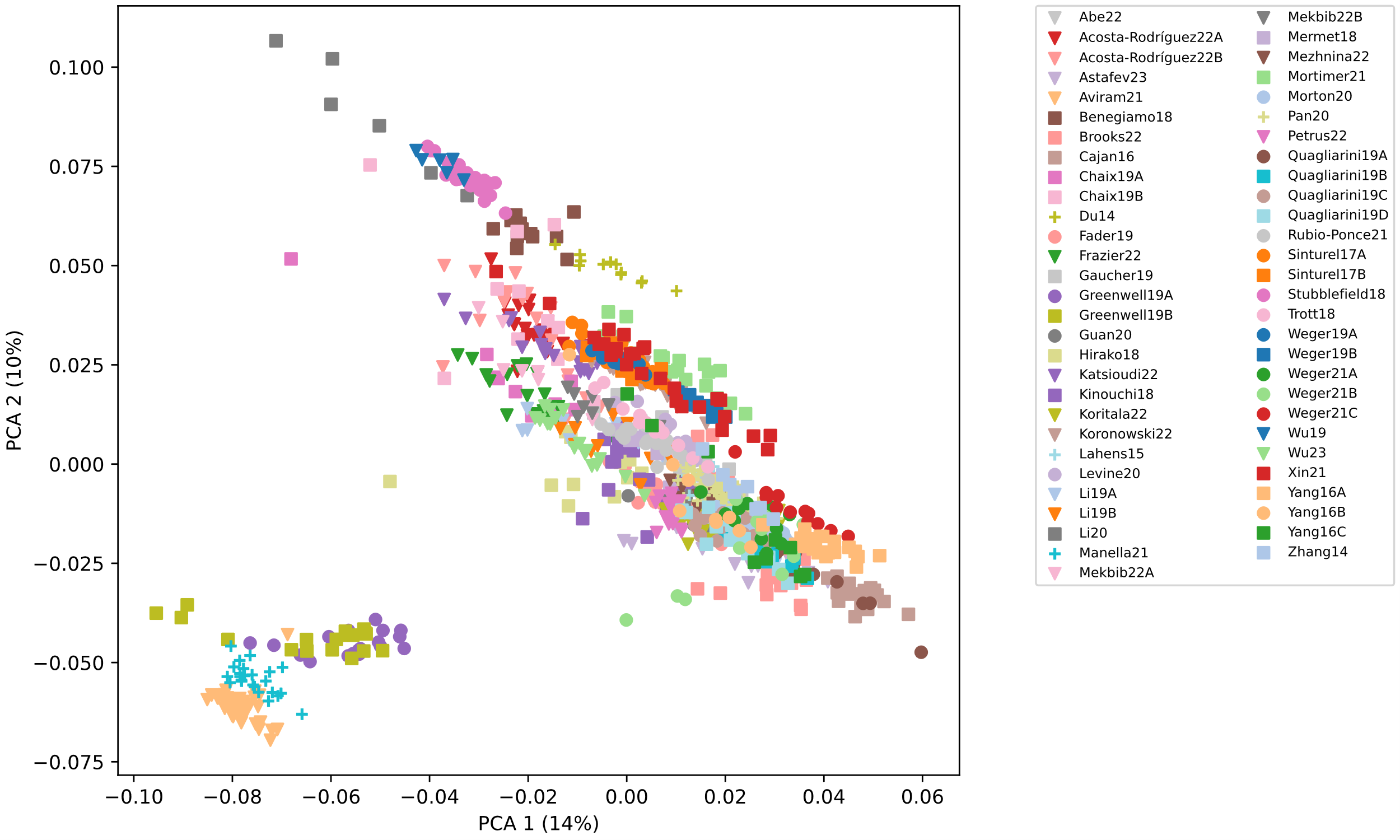 | 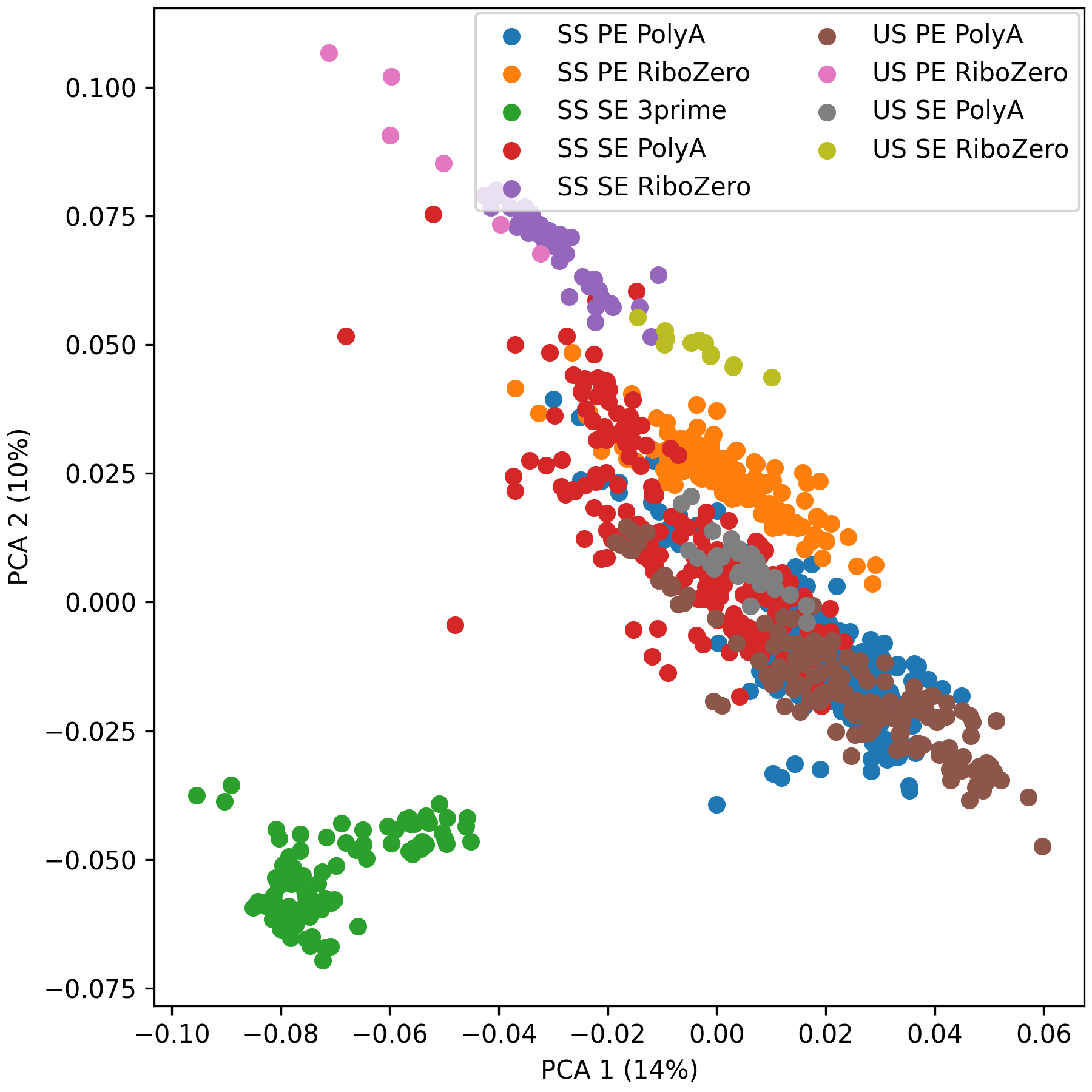 |
| --- | --- |

### Figure S 1 PCA of study data

Principal component analysis of RNA-seq from 907 mouse liver samples from 47 different timeseries. (left) PCA labelled by the timeseries each sample came from. (right) Labelled by the sequencing type of the study, revealing that the largest differences are due to technical choices. SS=strand-specific; US=unstranded; PE=paired-end; SE=single-end

| 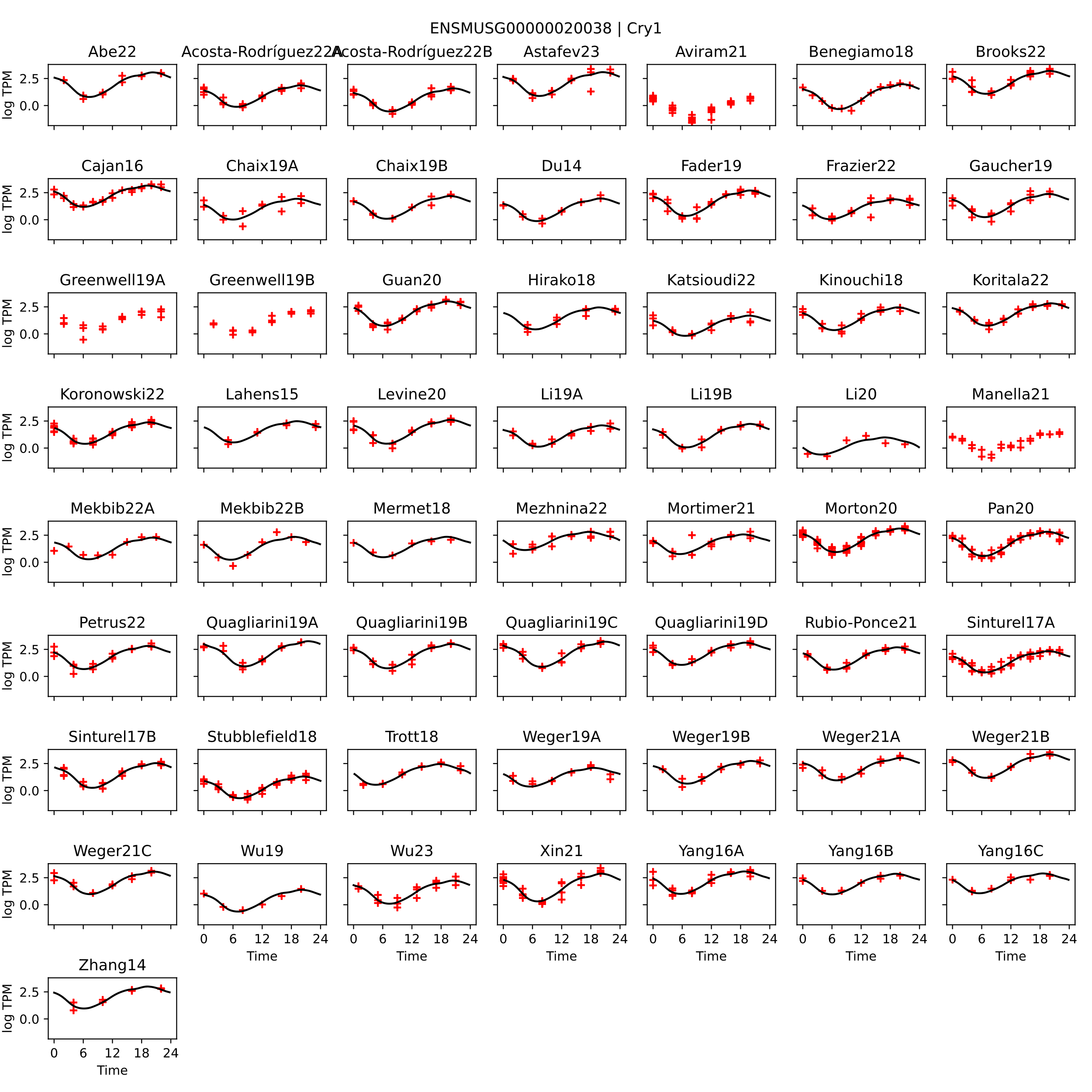 | 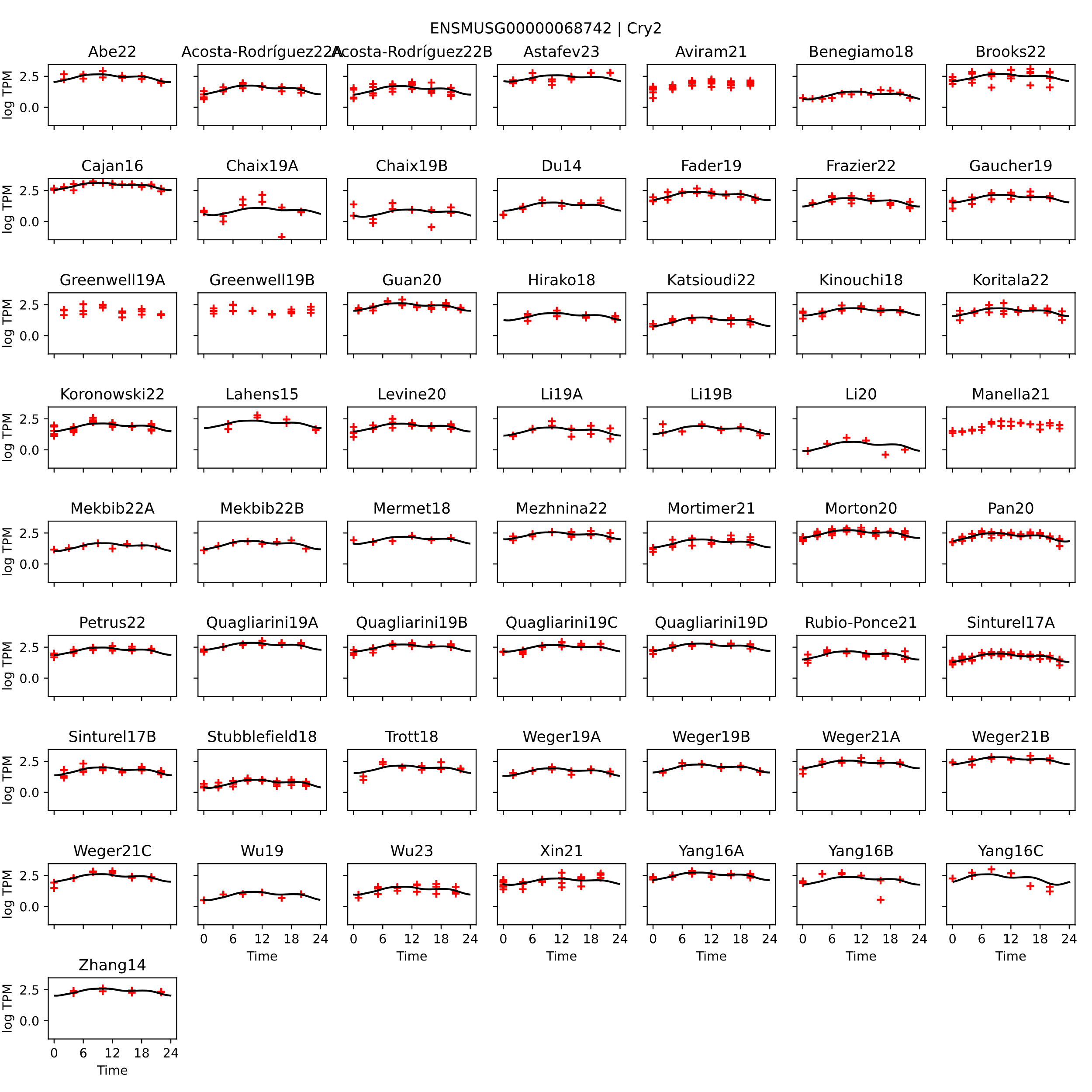 |
| --- | --- |
| 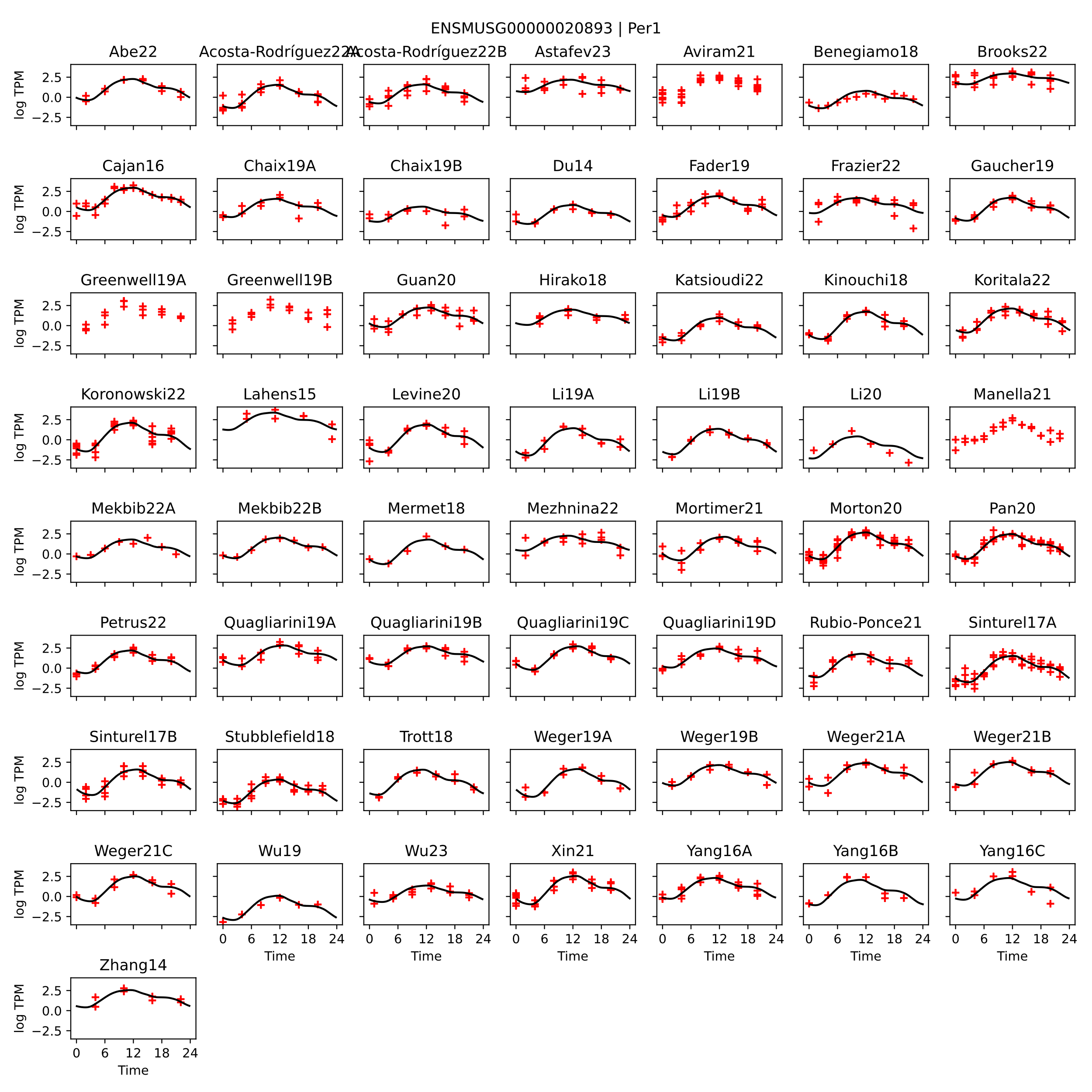 | 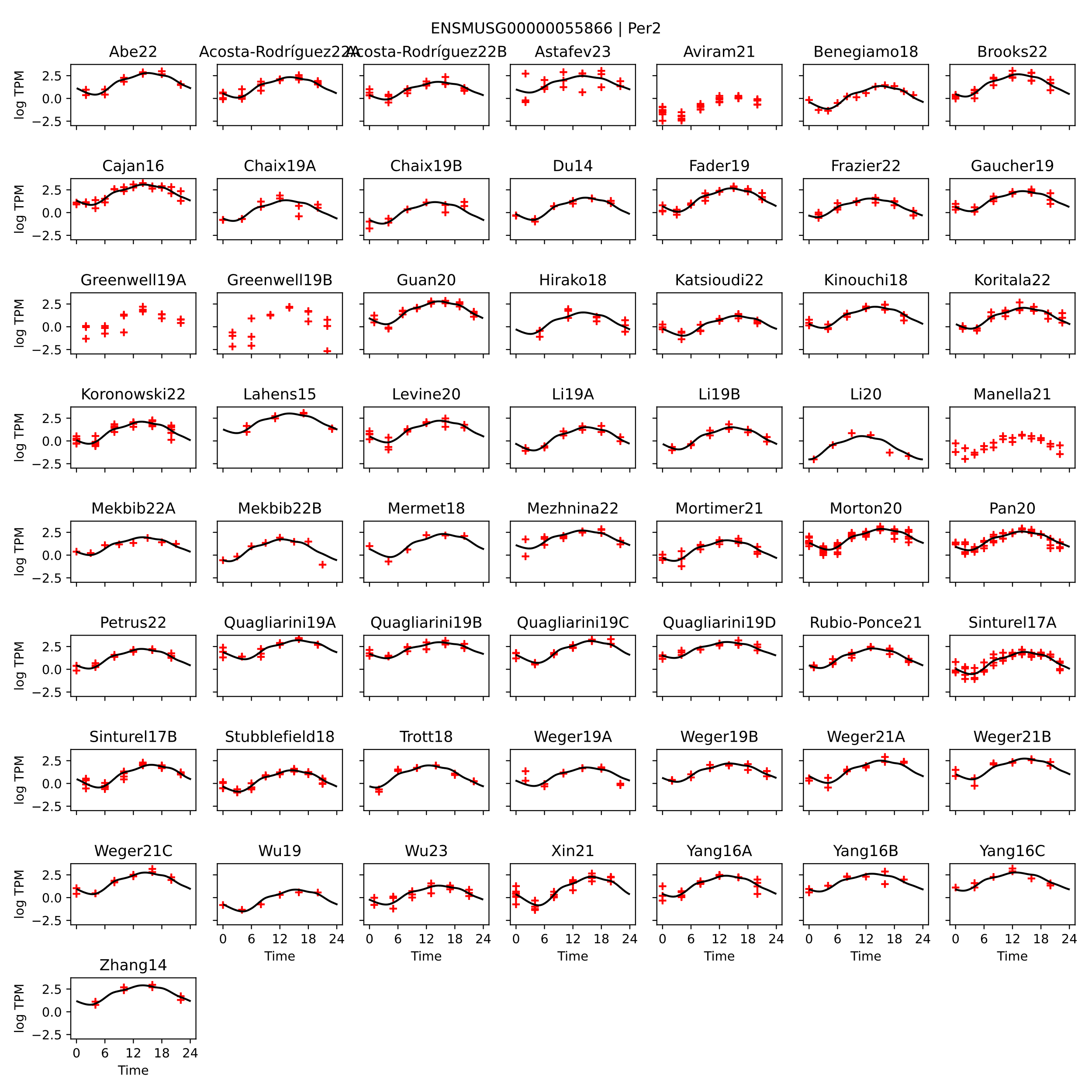 |
| 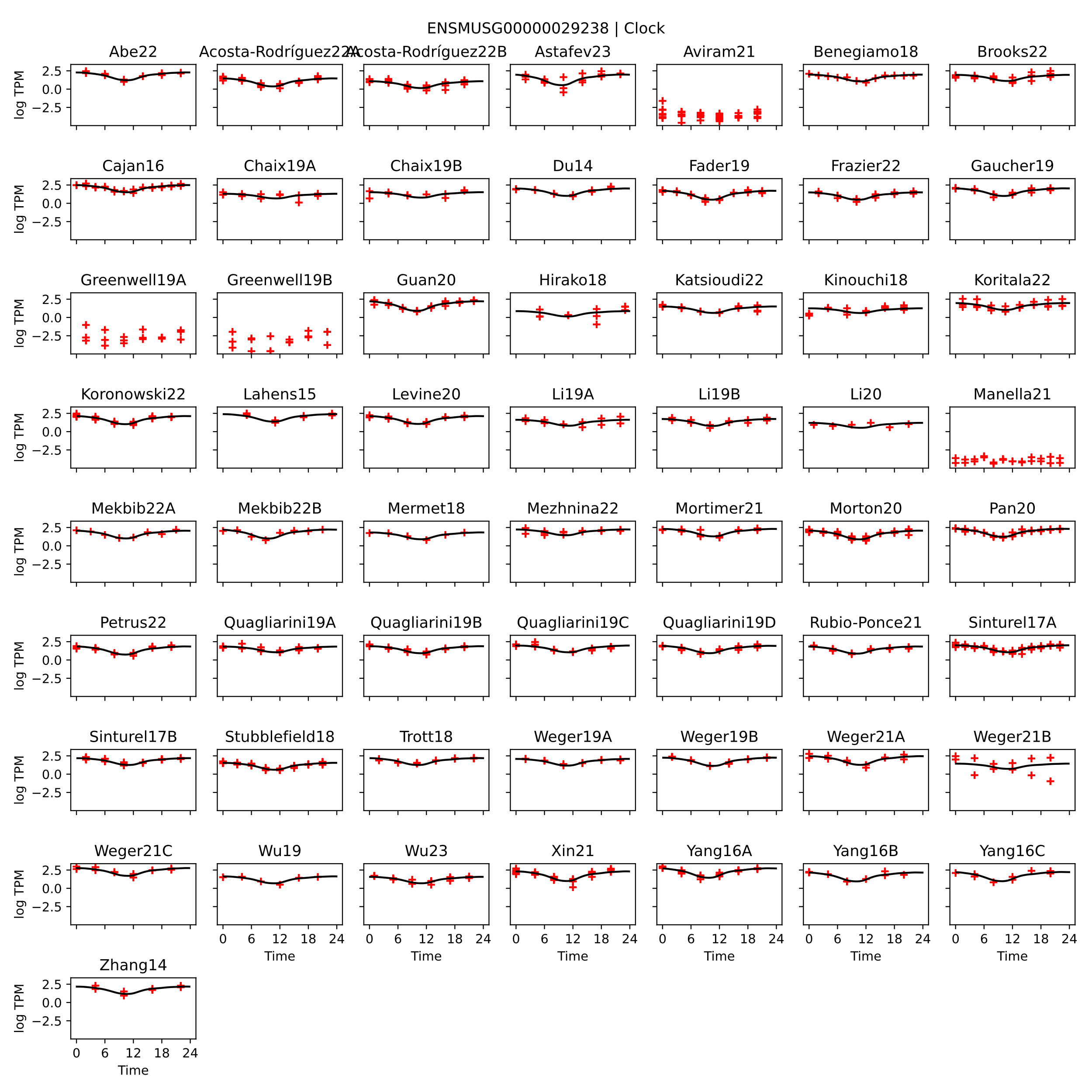 | 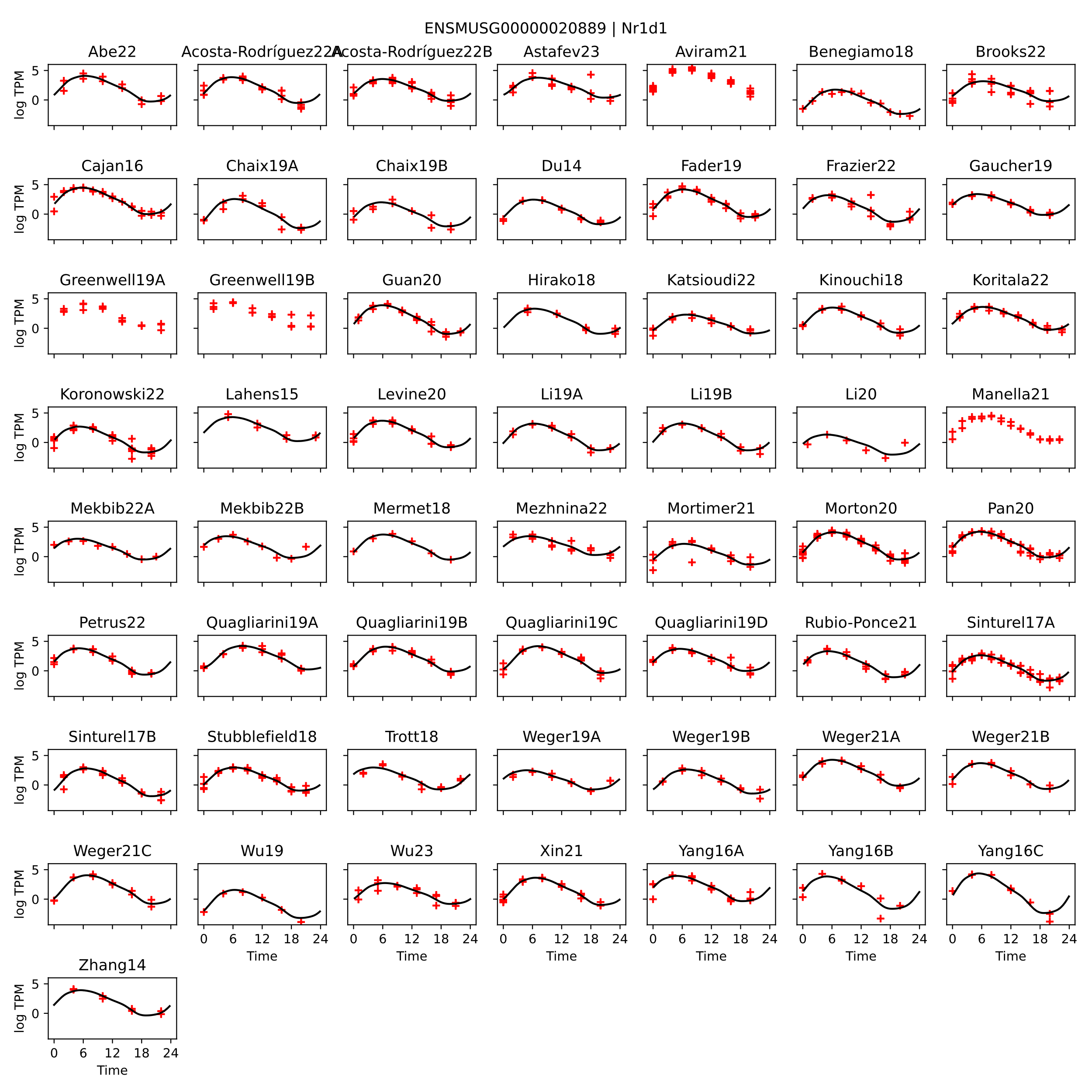 |

### Figure S 2 Core clocks gene expression profiles

Six core clock gene (Cry1, Cry2, Per1, Per2, Clock, and Nr1d1 from top left to bottom right) expression levels by time, in each of the 57 studies. Red dots are log TPM levels and black curves are the SIM fits (with four studies excluded due to unusual sequencing methodology). All clock genes show consistent shapes and phases across studies.


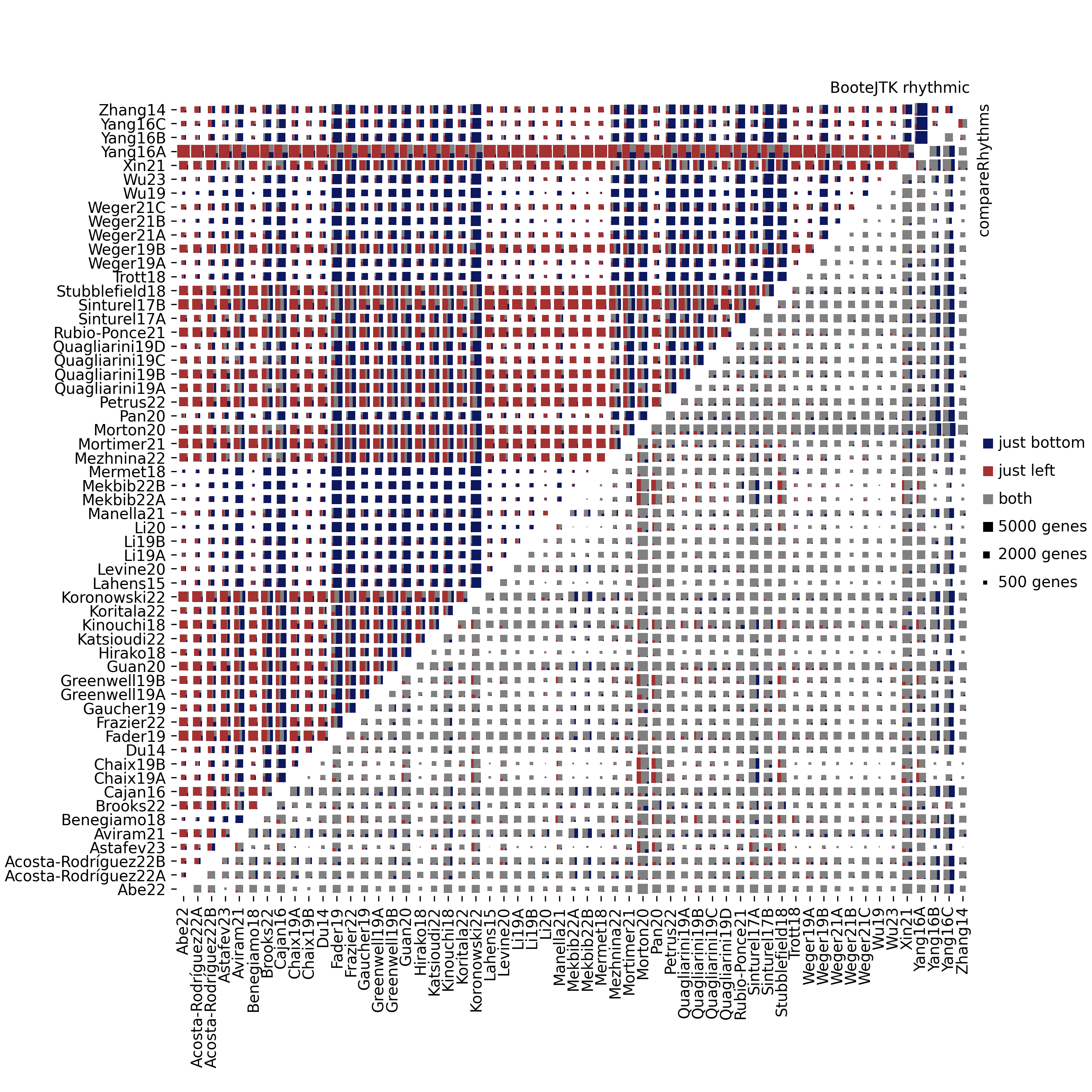


### Figure S 3 Rhythmic gene overlaps in BooteJTK and compareRhythms

Size of genes rhythmic common to both studies (in gray), unique in the row label study (red), or unique in the column label study (blue) shown for both BooteJTK (above diagonal) or compareRhythms (below diagonal). BooteJTK was run on each timeseries, and results were compared and genes significant at Benjamini-Hochberg q < 0.05 were considered rhythmic. Next, compareRhythm was run on every pair of studies which classified each gene as either loss, gain, change, or same rhythm between the two studies. Since expression values differ between studies making amplitudes changes unmeasurable, we considered genes to be rhythmic in both studies (possibly of differing amplitudes or phases) if they were identified as either same or change.


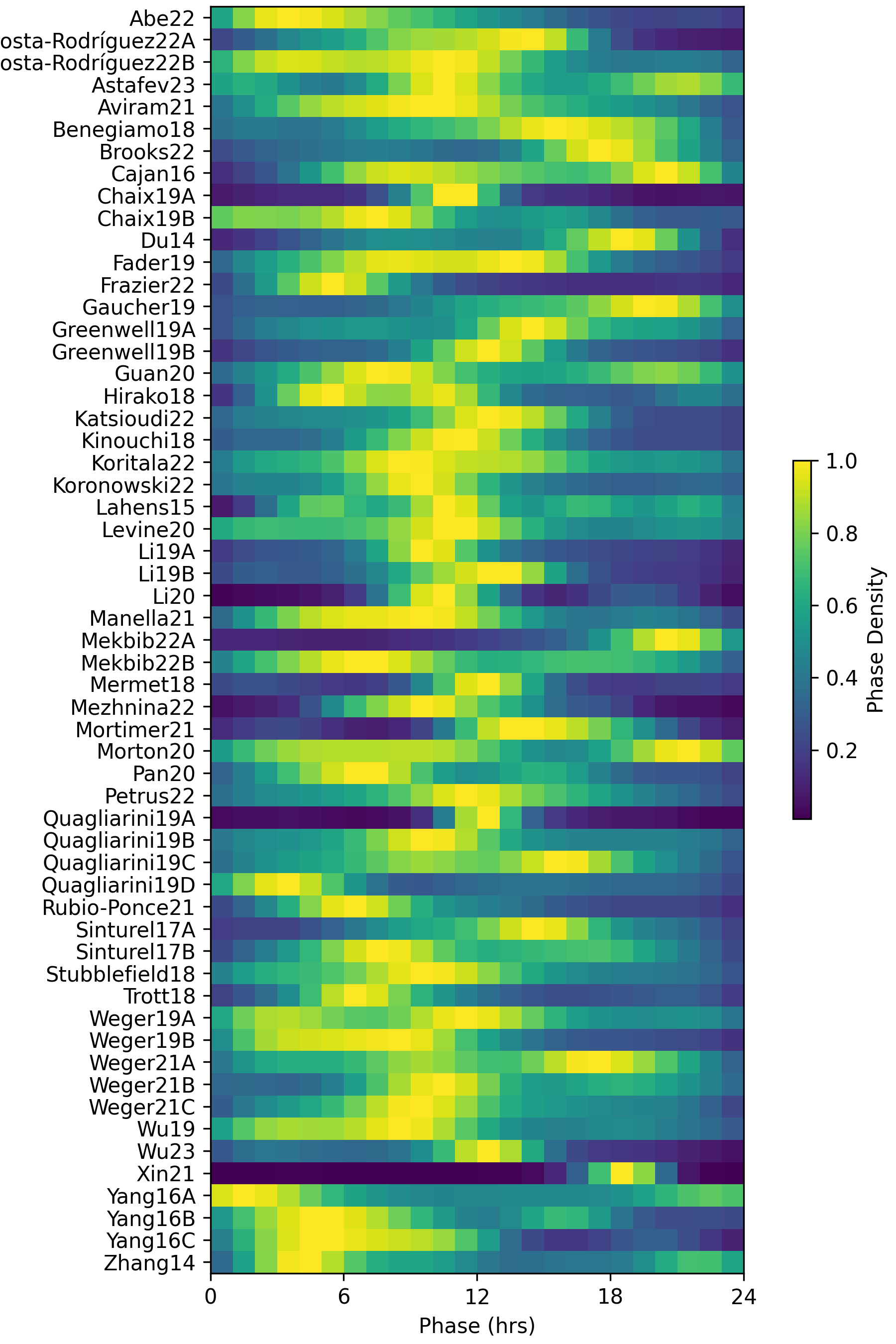


### Figure S 4 BooteJTK phase distributions

BooteJTK was run on each timeseries, and results were compared. Distributions of phases among genes identified rhythmic by BooteJTK (at a Benjamini-Hochberg Q-value < 0.05 from GammaP), showing notable inconsistency between studies. As under JTK, distributions are normalized to peak one, with the total number of genes identified as rhythmic shown in separate column.


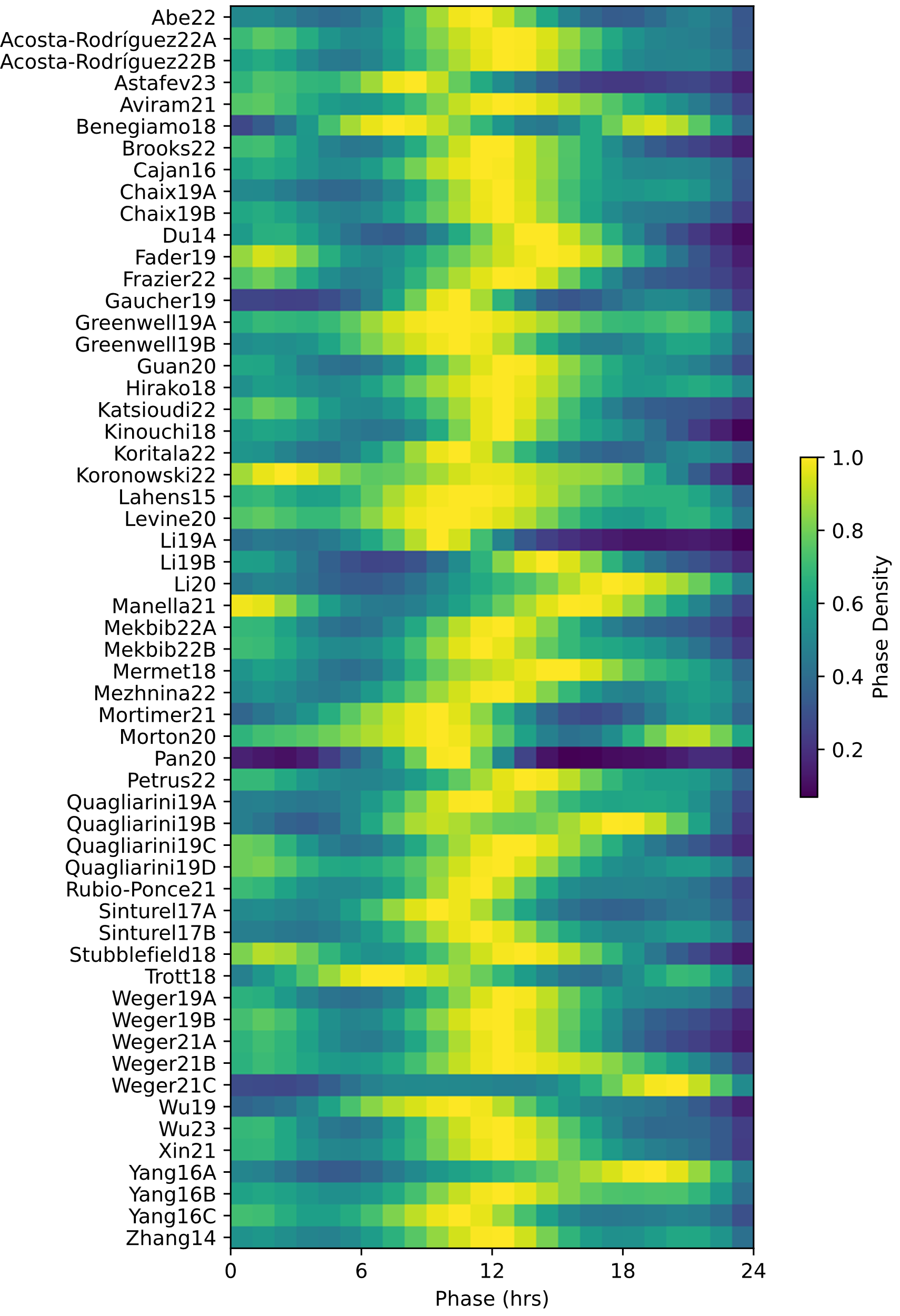


### Figure S 5 JTK Phase distribution in select genes

JTK_CYLCE was run on each timeseries, and results were compared. Distributions of phases among a fixed set of 525 genes that were consistently rhythmic (p < 0.05 in at least 35 studies). Compared to Figure 3, these show considerably higher consistency across studies Distributions are normalized to peak one.

| 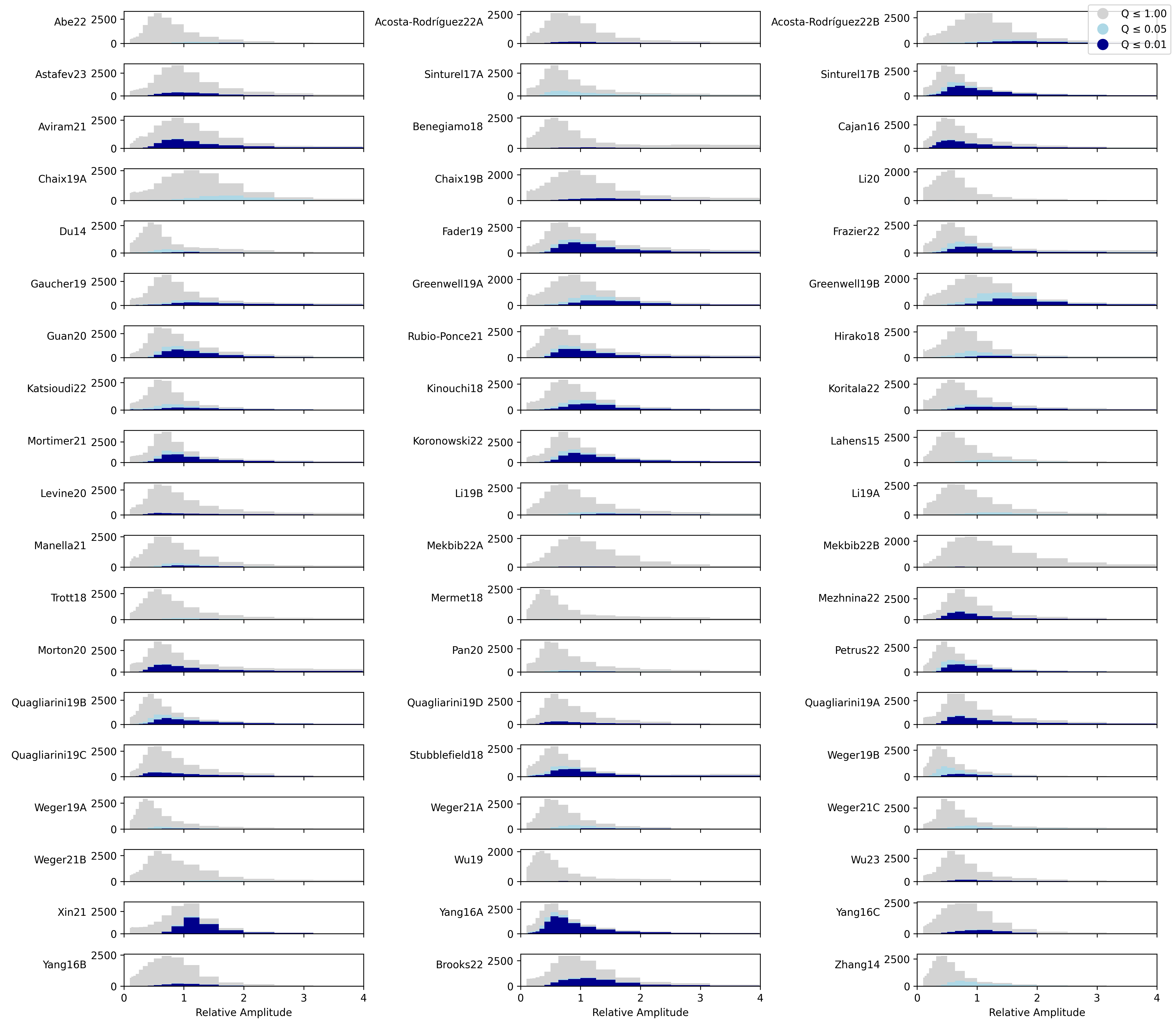 |
| --- |

### Figure S 6 BooteJTK amplitude distributions

Relative amplitude (calculated as (Max – Min)/(Mean+1)) distributions from BooteJTK results, among significantly rhythmic genes at three significance cutoffs (by color). Non-significantly rhythmic genes still have estimated amplitudes, so are included in this (in light gray).

| 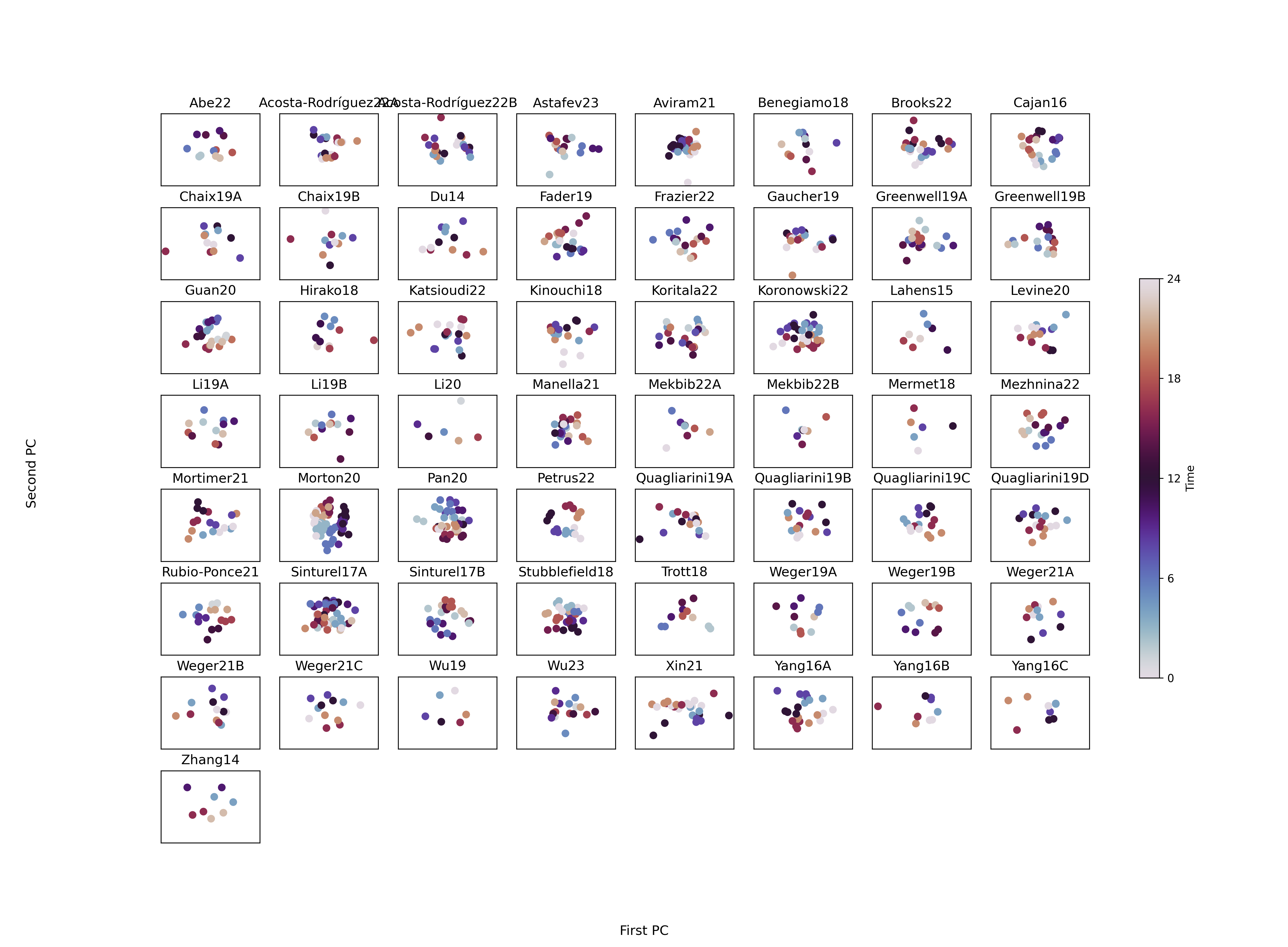 |
| --- |

### Figure S 7 Individual PCA

PCA was performed on each study individually and the top two components were plotted. Unlike JIVE, PCA components are unrelated in distinct studies, and these top two components do not reliably capture time variation within a study and are not comparable between different studies.


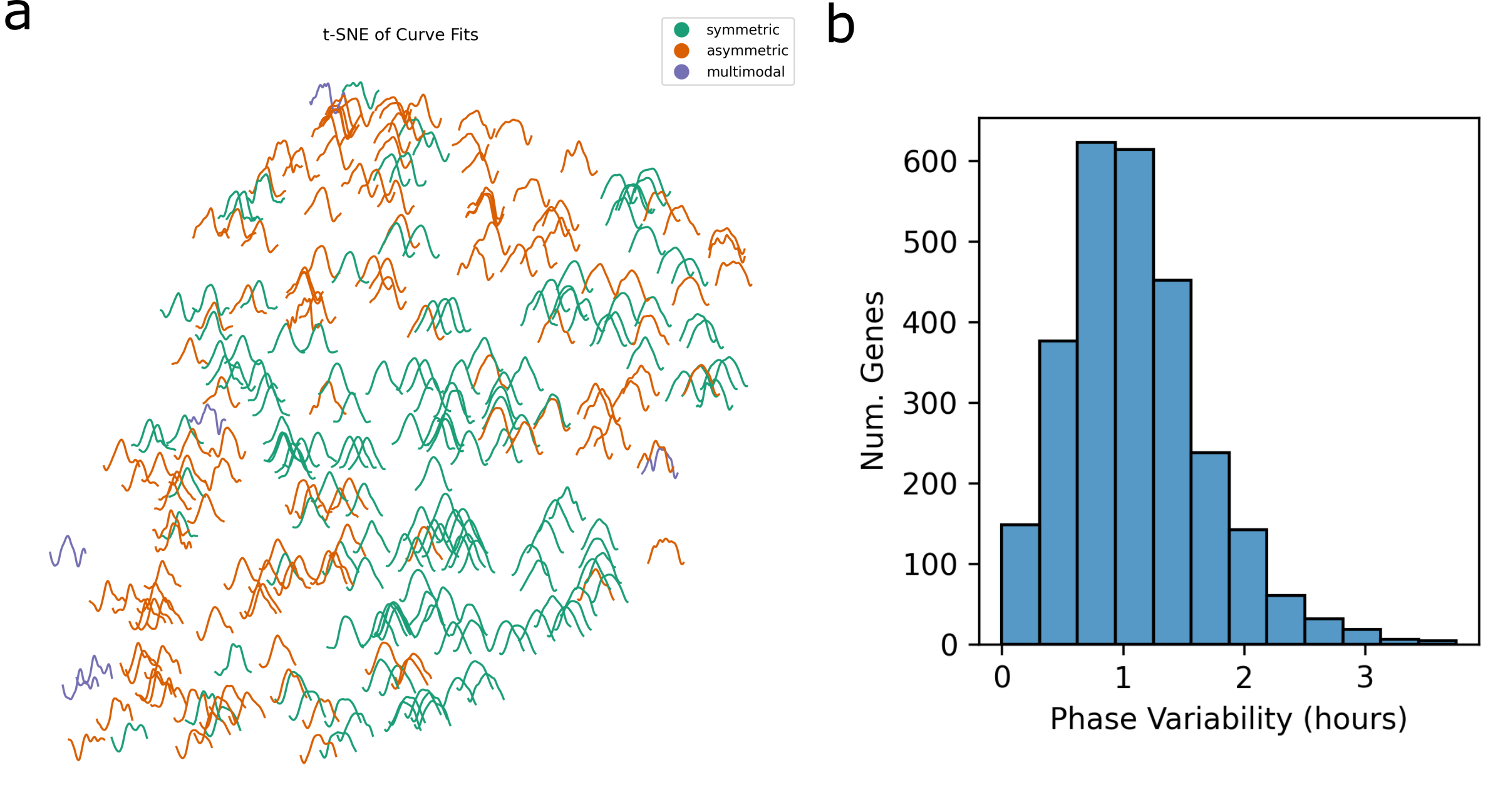


### Figure S 8 Shape invariant models

Shape invariant models were fit to each gene with random effects allowing variation of amplitude, mesor, and phase between studies. (a) In order to visualize the diversity of expression profiles, we plot a t-SNE of fit curves of identified rhythmic genes (300 randomly sampled genes shown). Fit curves were normalized to have the same peak, mean, and amplitude so that this plot captures only changes in overall shape. Genes were classified as symmetric (n= 1349 genes), asymmetric (n= 1291), or multimodal (n= 72) according to the fit curve. (b) Each study is fit a unique phase variable, pooling appropriately across studies. Histogram of the variability of the phases in the rhythmic genes, measured as the fit phi SD value, converted to hours by the inverse logistic function. The mean phase variability was 1.11 hours.

## Supplemental Files

### Table S1: Robustly Rhythmic Genes

List of genes with robustness core at least 35, indicating highly robust rhythms in most studies.

### Table S2: Highly Consistent Non-Rhythmic Genes

List of highly consistent, non-rhythmic genes (gene symbol and Ensembl gene ID) with low sample variance, for potential use as reference genes or “true nulls” in rhythmic analyses.

### File S1: Collected Data and Metadata

Compilation of all the quantified data, both as TPM and as counts, as well as sample metadata (study ID and time-of-day), study metadata (see Table 1) and results from JTK and SIM analyses. This data should be sufficient for future meta-analysis of these datasets.
